# Supplementary material for: Chromothripsis during telomere crisis is independent of NHEJ, and consistent with a replicative origin
Source: Genome Res. 2019 May;29(5):737–49. doi: 10.1101/gr.240705.118 (PMC6499312; doi:10.1101/gr.240705.118)
Supplement: Supplemental Material [file supp_gr.240705.118_Supplemental_file_1.zip › contigs/annotated_contigs/DB73/contig.5.DB73_length_863_mean_cov_13.1448435689.docx]

**DB73_length_863_mean_cov_13.1448435689**

```
GAACTTGTGAAAAAGAGAGTGGGGAATTTCGATCGATTTTGAACTTGCTTTGCTAGCTACAATCC|TT|CAAATATTGAATCTTTTAGG
 >chr3:81208254-81208321 - E=2e-28 p=4e-02                        >chr3:82287746-822878
CAGAGAATATACAATAAATATTTAAGTTGTATAAAAACCTAAGAGAGTAAAGAAAA|AAA|TAAAATTCTTTGAACAAGCAAATGCTGA
27 + E=7e-37 p=2e-02                                     >chr3:80297191-80297584 + E=2e
GGGATTTTGTTGCCACCAGGCATGCCTTGGAGGAACTCCTGAAGGAAGCAGTAAACACAGAAAAGAAAAATCATTACCAGCCACTACAA
-218 p=2e-02
AAACACACTAAAGTACACAGACCAATGACACTATGAAGTAACTACATTAACAAGTCTGCAAAATTAACCAGCTAGCACTATGATGACAG

GATCAAATTCACACATAACAACATTAACCTTAAATGTAAATGGGCAAAATGCTCCAATTGAAAGATACAGAATGGCAAGCTGATTAAAA

AGACAAGATTCATCAGTGTGCTTTTTTCAAGAGATACATTTCACATTCAAAGACACTCATAGGCTCAAAATAAACGGATGGAGGGGAAT

TTGCC|A|TTACTCCTTCACTTATGAAGCTTAGTTTGGCTGGATATGAAATTGTGGGTTGAAAATTCTTTTC|T|ATCTGCCAAGGACT
      >chr3:78726182-78726248 - E=8e-28                                >chr3:82288181
CCAAGTAAGTAGTTGATAGAAATAACAGTGCATAATGAGTAGACCAGTCTACATAGAGACTACAGGCCAAATTGGAACTATCTCAATCT
-82288440 + E=7e-138
TTGAAATTAAATTCAGGTAATAGGACATTGCGAGTTCCCAGGAGCCCATAAGAAGCAAGTAAAAAATTATCTCTGAAGTGAGACATAAT

CCTAGGCCTTGCATTATTTGTGTAATTTTTCAAGTACAGTGTGCTGCTAGCGAAAATAATAAAAAT|AAAA
```
